# Trying to identify who may benefit most from future vitamin D intervention trials: a post hoc analysis from the VITDAL-ICU study excluding the early deaths

**DOI:** 10.1186/s13054-019-2472-z

**Published:** 2019-06-04

**Authors:** Gennaro Martucci, Dayre McNally, Dhruv Parekh, Paul Zajic, Fabio Tuzzolino, Antonio Arcadipane, Kenneth B. Christopher, Harald Dobnig, Karin Amrein

**Affiliations:** 1 Department of Anesthesia and Intensive Care, IRCCS-ISMETT (Istituto Mediterraneo per i Trapianti e Terapie ad Alta Specializzazione), Palermo, Italy; 2 Faculty of Medicine, Division of Critical Care, Department of Pediatrics, Children’s Hospital of Eastern Ontario, University of Ottawa, Ottawa, Canada; 3 Critical Care, Birmingham Acute Care Research Group, Institute of Inflammation and Ageing, University of Birmingham, Birmingham, UK; 4 Division of General Anaesthesiology, Department of Anaesthesiology and Intensive Care Medicine, Medical University of Graz, Graz, Austria; 5 Research Office, IRCCS-ISMETT (Istituto Mediterraneo per i Trapianti e Terapie ad Alta Specializzazione), Palermo, Italy; 6 Brigham and Women’s Hospital, Harvard Medical School, Renal Division, Boston, MA USA; 7 Thyroid Endocrinology Osteoporosis Institute Dobnig, Graz, Austria; 8 Division of Endocrinology and Diabetology, Department of Internal Medicine, Medical University of Graz, Auenbruggerplatz 15, A-8036 Graz, Austria

**Keywords:** Vitamin D, Vitamin D responders, Epigenome, 28-day mortality, ICU mortality, Critical illness

## Abstract

**Background:**

Vitamin D supplementation has shown promise for reducing mortality in the intensive care setting. As a steroid prohormone with pleiotropic effects, there may be a lag between administration and observing clinical benefit. This secondary analysis of the VITdAL-ICU study sought to explore whether the effect size of vitamin D on mortality was different when study participants who died or were discharged early were excluded.

**Methods:**

The VITdAL-ICU study was a randomized, placebo-controlled trial in critically ill adults who received placebo or 540,000 IU cholecalciferol followed by monthly supplementation. The effect of vitamin D on 28-day mortality was evaluated after exclusion of participants who died or were discharged within 7 days from study drug administration, according to vitamin D concentrations on day 3, using a bivariate analysis adjusted for confounders and in a stepwise multiple analysis.

**Results:**

Of 475 study participants, 65 died or were discharged within the first 7 days. In the remaining 410 patients, vitamin D supplementation was associated with a reduction in 28-day mortality [OR 0.58 (95% CI 0.35–0.97) *p* value = 0.035]. The effect on mortality was not significant after adjusting for age, severity scores, female gender, chronic liver and kidney disease, COPD, diagnosis of the tumor, mechanical ventilation, and vasopressors at enrollment (all *p* > 0.05). In a multiple model, the mortality reduction by vitamin D supplementation did not remain independently significant [OR 0.61 (95% CI 0.35–1.05) *p* = 0.075].

Vitamin D metabolite response, in the treatment group, demonstrated that survivors at 28 days, had higher levels of 25-hydroxyvitamin D (34.4 vs 25.4 ng/ml, *p* = 0.010) and 1,25-dihydroxyvitamin D (107.6 vs 70.3 pg/ml, *p* = 0.049) on day 3. The increase of plasma metabolites after vitamin D oral supplementation, independent of the baseline value, was associated with lower odds of death [OR 0.48 (95% CI 0.27–0.87) *p* value = 0.016].

**Conclusions:**

High-dose vitamin D3 supplementation was associated with a reduction of 28-day mortality in a mixed population of critically ill adults with vitamin D deficiency when excluding patients who died or were discharged within 7 days after study inclusion. However, this survival benefit was not independently confirmed when adjusted for other factors strongly associated with mortality.

**Electronic supplementary material:**

The online version of this article (10.1186/s13054-019-2472-z) contains supplementary material, which is available to authorized users.

## Background

Vitamin D is a pre-hormone acting via its metabolite 1,25-dihydroxyvitamin D3, with genomic and non-genomic effects in most human tissues and cell types [1]. These wide-ranging biological effects include modulation of bone and muscle metabolism and a number of non-classical and pleiotropic effects. Such effects include immune function (anti-inflammatory function, energetic and redox homeostasis, innate immunity cell modulation) and cardiovascular modulation and may be essential to the development of, and recovery from, critical illness [2–5].

Vitamin D deficiency is common in critically ill patients [6]. Several large studies and meta-analyses have also found that vitamin D deficiency is associated with greater illness severity, morbidity, and mortality in both critically ill adult and pediatric patients [7–9]. The unanswered question is whether low vitamin D levels simply reflect a marker of greater disease severity or represent an independent and modifiable risk factor amendable to rapid normalization through loading dose supplementation [10, 11]. Large and well-designed randomized controlled trials (RCTs) in critically ill adults and children are still warranted in this field to clarify the topic [12, 13].

It is clear that the daily recommended low-dose administration of vitamin D (400 to 4000 IU daily) cannot rapidly restore 25 hydroxyvitamin D (25(OH)D) levels in acutely ill patients [14]. Several studies have adopted higher bolus dose supplementation (single loading dose from 50,000 to 600,000 IU) to rapidly restore vitamin D levels [15–19].

In addition to the need for high-dose vitamin D supplementation, it has to be considered that vitamin D works through endocrine, autocrine, and paracrine actions that activate a variety of rapid effects, but also a number of signaling and genomic pathways including epigenomic responses that probably need several days to become effective [20, 21]. Moreover, since the metabolism of vitamin D is complex and involves several organs, its action may be influenced by several conditions that may impair the oral supplementation activity, even at high dosage. Among others, those mainly reported are kidney and liver dysfunction, malabsorption, obesity, use of anti-seizure medications, and cardiopulmonary bypass [22–28]. Consequently, there may be a significant lag between providing the intervention and when benefits may be seen; thus, those unable to respond to supplementation rapidly may not have enough time to experience again in survival and likewise those recovering early probably do not recover because of vitamin D supplementation. Based on well-understood pathophysiology, one would anticipate that the lag would be at least 48 h (the time required for absorption and metabolism to 25(OH)D), but might well extend to a week while genomic effects are realized [1, 21]. The critical illness itself may also influence the availability of 25(OH) D, due to the liver and renal dysfunction or reduced vitamin D-binding protein (VDBP) and its conversion to the active metabolite 1,25-dihydroxyvitamin D (1,25(OH)D) [3]. While the actual absorption can be estimated by plasma levels, there are no validated biomarkers for evaluating late effects [29]. Taking into account these premises of time-dependent effects, it can be expected that patients dying early during the course of the ICU stay would probably not have enough time to fully benefit from cholecalciferol administration.

The VITdAL-ICU trial was the largest RCT to test the hypothesis that high-dose cholecalciferol supplementation in critically ill patients with vitamin D deficiency may rapidly restore plasma vitamin D concentrations and improve clinical outcomes [30]. While the primary endpoint of the length of stay was not significant, a secondary outcome showed that 28-day mortality was improved in patients with severe 25(OH) D deficiency who received high-dose cholecalciferol, but a clear separation of Kaplan Meier curves was seen only after 2 weeks.

In this secondary analysis, we hypothesized that exclusion of patients with early death or early discharge (less than 7 days after study drug administration) would better identify a cohort of patients in whom supplementation of vitamin D and increase in plasma 25(OH) D could be associated with a decrease in mortality. As secondary exploratory objectives, we explored how patient factors and comorbidities influenced the impact of vitamin D on 28-day mortality, including change in day 3 vitamin D levels.

## Methods

### VITdAL-ICU trial overview

The VITdAL-ICU trial was a randomized double-blind, placebo-controlled, single-center trial conducted in five ICUs of the Medical University of Graz (medical, neurological, cardiothoracic surgery, and 2 mixed-surgery units) [30]. The trial was approved by the Ethics Committee of the Medical University of Graz and the Austrian Agency for Health and Food Safety, and written informed consent was obtained directly from the patients or from a legal surrogate (NCT01130181). Inclusion criteria were 18 years or older, expected ICU stay of > 48 h, and vitamin D deficiency defined as a 25(OH) D level of 20 ng/ml or lower. Patients were considered not eligible in cases of severely impaired gastrointestinal function, other trial participation, pregnant or lactating women, do not resuscitate (DNR) order, hypercalcemia, sarcoidosis, or nephrolithiasis in the prior year.

Four-hundred and seventy-five patients (*n* = 475) constituted the intention-to-treat population for the primary analyses. Patients were assigned in a 1:1 ratio to the placebo arm (238) or a loading dose of 540,000 IU of vitamin D_3_ (237) and 5 monthly maintenance doses of 90,000 IU starting 28 days after initial load.

Patients were followed for 6 months, and outcome endpoints included the length of stay and mortality in the ICU at 28 days, in-hospital mortality, and mortality at 6 months. Although there was no difference in the primary outcome hospital length of stay, a non-statistically significant difference was suggested between groups for mortality.

### Patient selection for subgroup analysis and rationale for mortality outcome

We excluded from the study population, patients who died or were discharged within the first 7 days after study enrollment, and although the original trial reported on mortality at multiple time points, we selected 28 days mortality and 6-month mortality as the primary outcomes. The time-dependent mortality outcomes are more relevant in the perspective of exclusion criteria based on 7-day mortality/discharge, they are not influenced by local hospital discharge policies, and they are also related to the half-life of vitamin D metabolites that is expected to be between 7 and 21 days [31–34].

The excluded population was then compared with the included patients for the main baseline factors to raise the main indicators, which may further be considered warnings for patients unlikely to benefit from the vitamin D supplementation.

### Exploration of the effective role of vitamin D response in improved survival and confounding factors

To understand the impact of vitamin D supplementation on survival at 28 days, we compared the levels of vitamin D metabolites on day 3, 25-hydroxyvitamin D, and 1,25-dihydroxyvitamin D, among survivors and non-survivors.

To understand the effect of the increase of vitamin D levels from baseline, we tested the association between vitamin D increase and reduction in mortality in two ways. First, we examined the exposure as a binary variable, categorizing the respondents to vitamin D treatment in responders and non-responders according to the day 3 level of 25(OH)D: patients were classified as responders to vitamin D if they had a plasma level increase of > 10 ng/ml on day 3 after study drug administration, adjusted for the baseline value. This cutoff was arbitrarily decided based on previous studies that show how serum 25(OH) D levels increase by 10 ng/ml over 4 weeks for patients on daily 1000 IU vitamin D_3_, and on prior works showing differential outcomes with 25(OH) D levels categorized as < 10 ng/mL, 10–19.9 ng/mL, 20–29.9 ng/mL, and ≥ 30 ng/mL [35–40]. We also performed the same analysis including 25-hydroxyvitamin D on day 3 as a continuous variable to verify the potential reduction in mortality of every 1 ng/ml increase.

Finally, to understand if specific patient-related factors may impair the effect of vitamin D, we compared the association of different covariates with 28-day mortality and adjusted the effect of vitamin D supplementation for several covariates with interaction terms: age, gender, body mass index (BMI), type of admission (medical or neurological versus surgical), baseline value of 1,25-dihydroxyvitamin D, Charlson Comorbidity Index, simplified acute physiology score at admission (SAPS 2), therapeutic intervention scoring system (TISS), history of chronic kidney disease, chronic liver disease, COPD, ischemic heart disease or chronic heart failure, diagnosis of malignant disease, and the use of mechanical ventilation and vasopressors at enrollment.

### Statistical analysis

Baseline continuous variables are presented as means and standard deviation, while dichotomous and categorical variables are presented as number and percentage.

In the unadjusted analyses, chi-square and Fisher’s exact tests were used to compare mortality rates between placebo and vitamin D treatment groups. Multivariate logistic regression was used to investigate the relationship between vitamin D supplementation and 28-day mortality, including controlling for relevant covariates. Finally, multivariate logistic regression was used to report on predictors of early mortality, including baseline 25-hydroxyvitamin D and 1,25-dihydroxyvitamin D levels in response to treatment on day 3 among 28-day survivors and non-survivors, exploring the possible influencing factors.

To evaluate the survival benefit among responders and non-responders to vitamin D supplementation, a logistic regression adjusted for the baseline value of 25-hydroxyvitamin D was applied.

## Results

### Description of trial versus analytic cohort

From the intention-to-treat population (475 patients), 410 (86%) stayed in ICU more than 7 days and constituted the population of the secondary analysis (206 in the placebo arm and 204 in the treatment arm).

The baseline characteristics of included and excluded (*n* = 65, 43 deceased and 22 discharged early) patients, considering the exclusion criteria, were comparable and are illustrated in Table 1. Patients who died within 7 days, had a lower baseline level of 1,25-dihydroxyvitamin D (despite a comparably low level of vitamin D as inclusion criteria), and, as expected, had higher severity scores (SAPS 2 and TISS) and higher incidence of chronic kidney disease, were more frequently mechanically ventilated and more often on vasopressor support.

**Table 1:** Baseline differences between the included and excluded populations

	Included *N* = 410	Excluded *N* = 65	*P* value	Deceased < 7 days *N* = 43	Discharged < 7 days *N* = 22	*P* value
Age, years	64 ± 15	67 ± 12	0.06	69 ± 11.7	64.2 ± 12.8	0.13
Body mass index	27.2 ± 5.2	27.1 ± 5.7	0.94	26.9 ± 5	27.5 ± 6.4	0.69
25-Hydroxyvitamin D, ng/ml	13.2 ± 4.6	13.4 ± 5	0.67	12.9 ± 4.8	14.4 ± 5.4	0.26
1,25-Dihydroxyvitamin D, pg/ml	42 (38–46)	47 (36–58)	0.36	29.3 ± 21.5	83.1 ± 56.2	< 0.01
Charlson Comorbidity Index	3 (2.8–3.2)	3.4 (2.9–3.9)	0.25	3.6 ± 2	2.9 ± 1.9	0.23
SAPS 2	32.7 ± 15.4	36.6 ± 15	0.06	39.4 ± 15	31 ± 14	0.04
TISS	38 ± 7.7	37 ± 8.8	0.36	41.3 ± 7.3	28.8 ± 4.6	< 0.01
Male/female gender, *N*/*N*	267/143	42/23	0.94	30/13	12/10	0.22
Chronic kidney disease, *N* (%)	113 (27.5)	21 (32.3)	0.43	18 (41.8)	3 (13.6)	0.02
Surgical/medical and neuro admission *N*/*N*	240/170	14/51	< 0.01	12/31	2/20	0.08
Chronic liver disease, *N* (%)	61 (14.9)	10 (15.4)	0.85	7 (16.3)	3 (13.6)	0.78
COPD, *N* (%)	77 (18.8)	10 (15.4)	0.61	8 (18.6)	2 (9.1)	0.31
Ischemic heart disease—chronic heart failure, *N* (%)	229 (55.8)	26 (40)	0.59	26 (60.5)	8 (36.4)	0.07
Malignant disease, *N* (%)	34 (8.3)	4 (6.2)	0.55	2 (4.7)	2 (9.1)	0.48
Mechanical ventilation at enrollment, *N* (%)	268 (65.0)	34 (52.0)	0.09	34 (79.1)	0	< 0.01
Use of vasopressor at enrollment, *N* (%)	203 (49.5)	32 (49.2)	0.44	32 (74.4)	0	< 0.01

Continuous variables are presented as value ± standard deviation or median (95% confidence interval), binary variables as number, and percentage

*Responder to vitamin D treatment: patients with an increase of 25-hydroxyvitamin D plasma levels on day 3 after enrollment of at least 10 ng/ml

*SAPS 2* simplified acute physiology score 2, TISS Therapeutic intervention scoring system

### Effect of vitamin D supplementation on mortality

In this selected population, vitamin D supplementation reduced 28-day mortality in patients who survived over the first 7 days and stayed more than 7 days: at 28-day, 30 patients (14.7%) were deceased in the treatment arm, and 47 patients (22.8%) in the placebo arm [OR 0.58 (95% CI 0.35–0.97) *p* value = 0.035]. This reduction was not statistically significant for 6-month mortality [OR 0.67 (95% CI 0.44–1.01) *p* = 0.055]. In the intention-to-treat population, the OR was higher and did not reach statistical significance [OR 0.76 (95% CI 0.53–1.09) *p* value = 0.14]. All the odds ratios for mortality outcomes in the selected population and the intention-to-treat population are reported in Additional file 1: Tables S1 and S2.

### Vitamin D metabolites, their response, and mortality

First, we compared 25OHD and 1,25-dihydroxyvitamin D levels at baseline and on day 3 after admission both in the treatment and in the placebo group (Table 2). 25-Hydroxyvitamin D levels at day 0 were low in both the vitamin D and placebo group, with no difference between survivors and non-survivors at 28 days. Similarly, 1,25-dihydroxyvitamin D at day 0 showed a low level in both the vitamin D and placebo group.

**Table 2:** Vitamin D metabolite level at baseline and day 3 after enrollment

Vitamin D metabolite	Survivors at 28 days Mean (CI 95%)	Non-survivors at 28 days Mean (CI 95%)	*p* value
Vitamin D supplementation group, day 0			
25-Hydroxyvitamin D, ng/ml	12.9 (12.2–13.6)	13.0 (11.3–14.7)	0.869
1,25-Dihydroxyvitamin D, pg/ml	44.3 (37.1–51.4)	38.3 (21.9–54.7)	0.527
Placebo group, day 0			
25-Hydroxyvitamin D, ng/ml	13.6 (12.9–14.4)	12.9 (11.6–14.4)	0.382
1,25-Dihydroxyvitamin D, pg/ml	42.9 (36.3–49.4)	34.6 (25.9–43.3)	0.133
Vitamin D supplementation group, day 3			
25-Hydroxyvitamin D, ng/ml	34.4 (31.7–37.2)	25.4 (20.2–30.6)	0.010
1,25-Dihydroxyvitamin D, pg/ml	107.6 (92.7–122.6)	70.3 (36.0–104.7)	0.056
Placebo group, day 3			
25-Hydroxyvitamin D, ng/ml	13.9 (13.2–14.7)	13.9 (12.1–15.7)	0.948
1,25-Dihydroxyvitamin D, pg/ml	50.7 (42.2–59.2)	37.4 (26.5–48.4)	0.059

**Fig. 1:**
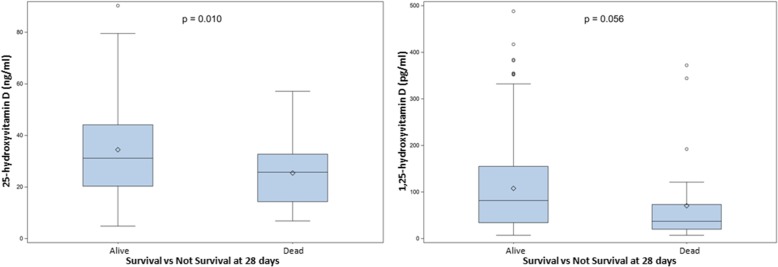
Difference among survivors and non-survivors at 28 days, in the treatment group, on day 3 values of 25-hydroxyvitamin D and 1,25-dihydroxyvitamin D

At day 3, in the placebo group, no difference was seen in 25OHD between survivors and non-survivors, while for 1,25-dihydroxyvitamin D the difference was less evident than at baseline (*p* = 0.059). In the treatment group, on day 3 (when the absorption of the loading dose is supposedly complete), 25OHD levels were significantly higher in survivors than in non-survivors, while 1,25-dihydroxyvitamin D showed a higher level in survivors but without reaching statistical significance (Fig. 1).

Finally, looking at the association of individual vitamin D response and outcome, we explored whether an increase in 25OHD was associated with improved chances of survival according to the category responders described in the “Methods” section. Moreover, in the placebo group, there were no patients with a spontaneous increase in 25OHD, while in the vitamin D_3_ study arm, 133 patients out of 204 (65%) were responders. Responders had a > 50% mortality reduction [OR 0.48 (95% CI 0.27–0.87) *p* value = 0.016]. This association was still significant after correcting for the baseline 25OHD.

Baseline differences between responders and non-responders are illustrated in Table 3. Non-responders were characterized by higher baseline severity expressed by higher TISS score and higher rate of mechanical ventilation and vasopressor use, as well as higher rate of surgical admission and previous chronic liver disease.

**Table 3:** Different baseline characteristics of patient responder and non-responder to vitamin D supplementation on day 3 after study drug administration. Odds ratios for 28-day mortality for vitamin D supplementation in a bivariate analysis with an interaction term for the covariates. If the interaction term was not significant, the odds ratio of treatment adjusted for covariate without interaction term was reported

	Vitamin D supplementation group (*N* = 204)*		
	Responder (*N* = 133)	Non-responder (*N* = 71)	*p* value
Age, years	64 ± 16	61 ± 13	0.19
Body mass index	27.3 ± 4.7	27.2 ± 4.9	0.87
25-Hydroxyvitamin D, ng/ml	13.2 ± 4.5	12.3 ± 4.8	0.19
1,25-Dihydroxyvitamin D, pg/ml	48 (40–57)	34 (24–44)	0.05
Charlson Comorbidity Index	2.8 (2.3–3.2)	3 (2.5–3.5)	0.59
SAPS 2	33 (30–36)	29 (26–32)	0.04
TISS	36 (35–37)	41 (39–42)	< 0.01
Male/female gender, *N*/*N*	86/47	45/26	0.86
Chronic kidney disease, *N* (%)	35 (26.3)	18 (25.4)	0.88
Surgical/medical and neuro admission *N*(%)/*N*(%)	64 (48)/69 (52)	55 (77)/16 (33)	< 0.01
Chronic liver disease, *N* (%)	13 (9.8)	16 (22.5)	0.01
COPD, *N* (%)	27 (20.3)	10 (14)	0.27
Ischemic heart disease—chronic heart failure, *N* (%)	67 (50.4)	36 (50.7)	0.96
Malignant disease, *N* (%)	13 (9.8)	6 (8.5)	0.76
Mechanical ventilation at enrollment, *N* (%)	78 (58.6)	55 (77.5)	0.02
Use of vasopressor at enrollment, *N* (%)	57 (42.9)	47 (66.2)	< 0.01

Definition of responder to vitamin D supplementation: plasma level increase of > 10 ng/ml on day 3 after study drug administration

*The analysis is performed just in the treatment group since in the placebo group, there were no patients classified as responders according to our criteria

The association between individual response to vitamin D supplementation and mortality reduction remained significant if change in 25OHD levels was included in the model as continuous variable: for every 1 ng/mL increase in 25OHD on day 3, the odds of death at 28 days were significantly reduced [OR 0.96 (95% CI 0.94–0.98) *p* value < 0.001].

### Impact of covariates on vitamin D supplementation and mortality

**Fig. 2:**
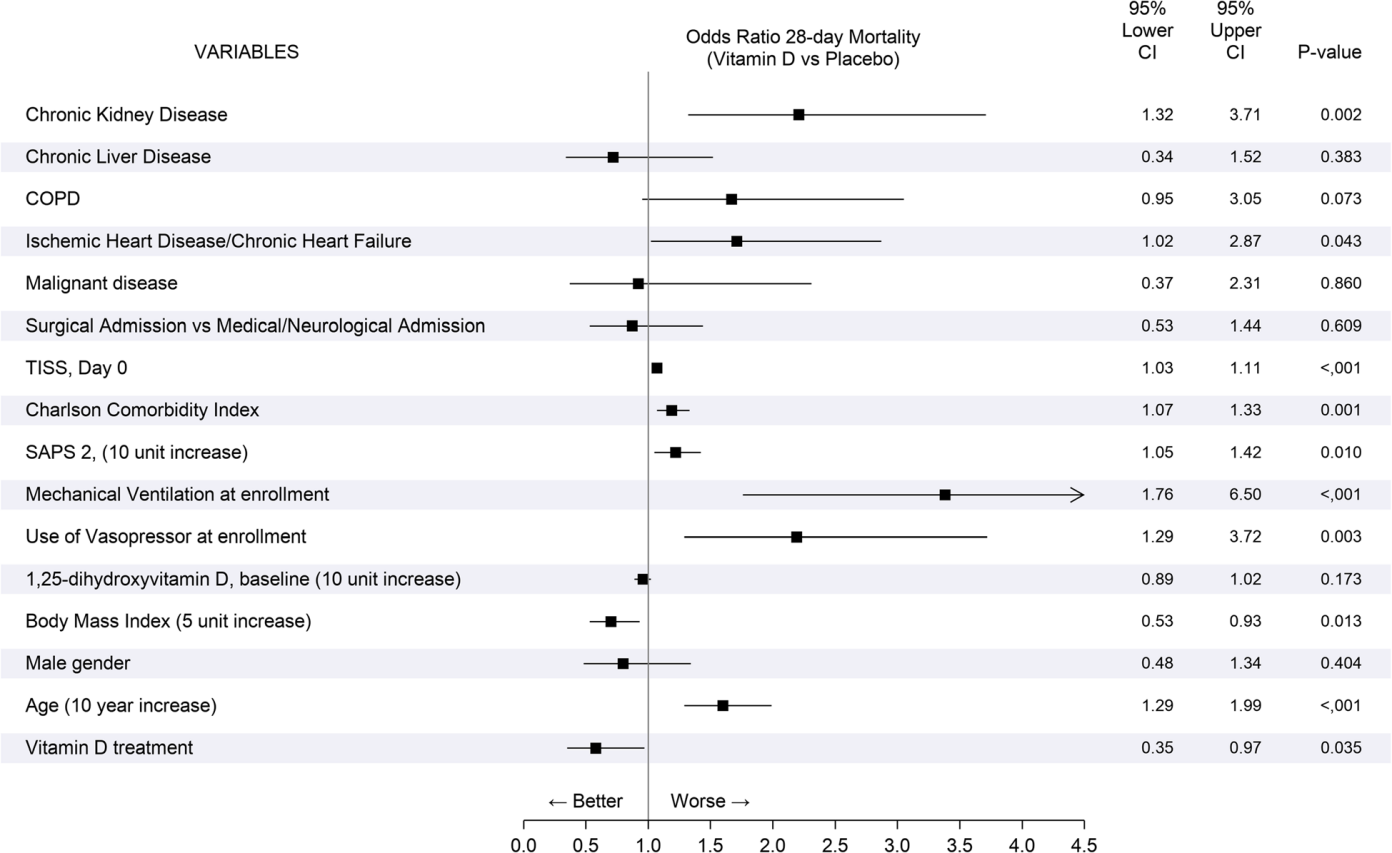
Univariate analysis. The odds ratio for 28-day mortality, for vitamin D supplementation, and for several clinically relevant covariates

To understand the patient-related factors associated with mortality at 28 days, we generated the odds ratio for vitamin D supplementation and for several covariates (Fig. 2). The most important factors associated with mortality were the need for vasopressors from the beginning of ICU admission, older age, higher severity scores (Charlson Comorbidity Index, SAPS 2, and TISS) and comorbidities such as chronic kidney disease, previous diagnosis of ischemic heart disease, or chronic heart failure. Vitamin D was associated with survival [OR 0.58 (95% CI 0.35–0.97), *p* value = 0.035] as well as higher BMI.

**Fig. 3:**
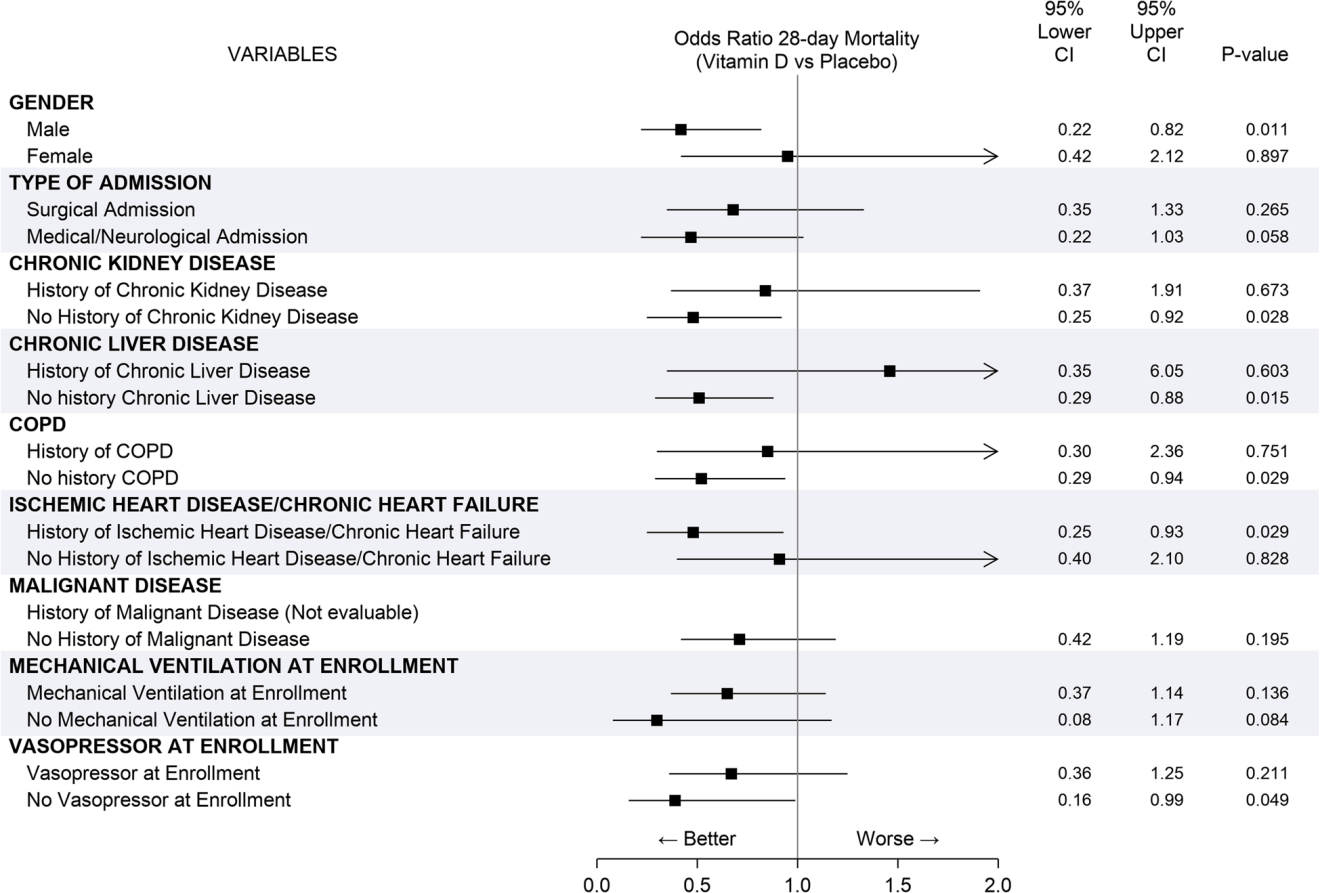
Patient factors influencing the impact of the vitamin D supplementation on late death bivariate analysis for categorical variables. The interactions were reported with a distinct row for each modality of the factor

In addition, we analyzed how specific patient factors influence the impact of the vitamin D supplementation on late death. Bivariate logistic regression evaluating continuous covariates (e.g., age, BMI, baseline 1,25 dihydroxyvitamin D levels), despite interaction terms did not identify any that were statistically significant, showed how age, Charlson Comorbidity Index, and SAPS 2 were able to reduce the impact of vitamin D supplementation on mortality, reducing the statistical significance of the odds ratio (Table 4). Moreover, as shown in Fig. 3, evaluation of categorical variables showed that vitamin D administration may have less clinical benefit in ICU female patients affected with chronic liver disease and chronic kidney disease as well as in patients with a higher severity of illness at admission (as showed by the impact of vasopressor use at enrollment).

**Table 4:** Odds ratios for 28-day mortality for vitamin D supplementation in a bivariate analysis with an interaction term for the covariates. If the interaction term was not significant, the odds ratio of treatment adjusted for covariate without an interaction term was reported

Adjusting factors	Odds ratio Vitamin D vs. placebo	95% confidence limits	*p* value	Interaction term *p* value
Age	0.60	0.34–1.06	0.082	0.997
Body mass index	0.56	0.33–0.95	0.032	0.385
1,25-Dihydroxyvitamin D, baseline	0.59	0.35–0.98	0.044	0.542
Charlson Comorbidity Index	0.63	0.37–1.06	0.081	0.552
SAPS 2	0.61	0.36–1.02	0.061	0.702
TISS day 0	0.58	0.34–0.99	0.049	0.800

In a stepwise multiple logistic regression of 28-day mortality, adding the interaction term for every variable, the factors that remained associated with change in mortality with statistical significance were age [OR 1.05 (95% CI 1.02–1.07) *p* < 0.001], body mass index [OR 0.92 (95% CI 0.87–0.98) *p* = 0.010], Charlson Comorbidity Index [OR 1.14 (95% CI 1.01–1.29) *p* = 0.045], and the absence of mechanical ventilation at enrollment [OR 0.31 (95% CI 0.14–0.69) *p* = 0.004]. Evaluating the treatment in the model, vitamin D supplementation showed a trend toward reduction in 28-day mortality without reaching statistical significance [OR 0.61 (95% CI 0.35–1.05) *p* = 0.075].

## Discussion

In this post hoc analysis of the VITdAL-ICU study, we aimed to better identify who may benefit most from future vitamin D intervention trials, based on the theoretical model that because of the time-dependent effects of vitamin D, some patients may be “too ill” or “too well” and vitamin D may be unable to modify the clinical trajectories in these subgroups. Given the substantial cost and time of RCTs, it would be helpful to better predict which patients may benefit most and focus on these. Excluding patients who died (“too ill”) or were discharged (“too well”) within 7 days from study drug administration, we found a significant reduction in 28-day mortality with a loading dose of cholecalciferol supplementation. Furthermore, we demonstrated that a more robust increase in 25-hydroxyvitamin D and 1,25-dihydroxyvitamin D on day 3 was associated with a survival benefit. Finally, we highlighted how multiple patient characteristics (female gender, surgical admission, chronic kidney disease, and chronic liver disease) significantly reduced the impact of vitamin D supplementation on mortality, and the survival effect was not independent when adjusted for well-known factors strongly influencing mortality.

Vitamin D is a steroid precursor hormone and, after conversion into the active form, modulates the expression of various matrix metalloproteinasis growth factors and cytokines involved in the inflammatory response [41, 42]. Vitamin D level in humans is the result of endogenous production via the exposure to UV-B from sunlight and of oral intake as food or supplementation, both reduced or abolished in critically ill patients [43]. In cases of systemic injury, there is an enhanced conversion of 25-hydroxyvitamin D into its active form 1,25-dihydroxyvitamin D to meet the increased tissue demand and an enhanced catabolism of metabolites [44]. Moreover, critically ill patients are often hemodiluted and, as a consequence of inflammation, have lower protein production, with consequent lower albumin and VDBP levels that contribute to lowering the plasma levels of vitamin D [6, 36, 45]. In such a context, there are several factors that may be associated with vitamin D deficiency, such as age, BMI, skin pigmentation, a gastrointestinal disease with malabsorption, and liver and renal disease [9, 14, 28, 46].

Because of these possible interactions, its metabolism is complex and needs time for the end-organs and target cells to respond through genomic activation and induction of metabolic pathways [21]. In the more severely ill patients, it is possible that the time to await these vitamin D actions to occur is not sufficient as the trajectory of acute illness that finally leads to multi-organ dysfunction and death has already commenced.

The rationale of the trials conducted in recent years on vitamin D supplementation in critically ill patients was that large loading doses would restore the plasma vitamin D concentration, leading to a survival benefit [13]. But in some patients, as suggested by our data, this does not happen, giving apparently negative results, which were challenged by excluding patients who died too early, and in those in whom there was not enough time for effective supplementation [47]. In addition to reduce the potential bias of selective patients’ exclusion, we excluded also patients discharged early (within the same time frame) with the hypothesis that such patients would have recovered likely not because of vitamin D supplementation. Our data show a reduction in 28-day mortality, while the outcomes without a defined time point (hospital and ICU mortality) do not show a significant benefit. This is most likely due to multiple confounders that may affect the length of hospital stay and severity of illness [48]. Similarly, the excluded deceased patients were also characterized by higher severity scores (and consequently also with higher mortality risk) and we can hypothesize that when in the acute phase there is severe multi-organ impairment, vitamin D may be ineffective or less effective than in patients with a better short-term mortality risk profile. This is confirmed also by the characteristics of patients able to increase their plasma vitamin D levels 3 days after vitamin D high-dose supplementation. Also, the increase of vitamin D level was negatively influenced by baseline severity and was less significative in case of surgical admission and pre-existent liver disease. This information is important for the methodology of ongoing and potential prospective multicenter studies of vitamin D replacement in acute care. Our data suggests considering excluding patients with very high baseline severity scores or who die in the early course of the ICU stay, or at least to plan in advance subgroup analyses excluding patients with early deaths and early discharge.

In our study, we investigated patient variables that may influence the responses to vitamin D supplementation [49, 50]. This is a relevant field of investigation in healthy and critically ill patients, but in our cohort, there was evidence that patients who were responders have a better chance of surviving [51]. This survival benefit is present if we consider patients with a relevant increase (more than 10 ng/ml), but also as a continuous variable and regardless of the baseline concentration. However, we are currently only able to monitor the plasma levels of 25(OH) D and 1,25(OH)_2_D, but not the real effect of supplementation on the complex vitamin D metabolism and axis. Results from studies on metabolomics will probably provide a better understanding of this area but require further evaluation in critical care populations [29, 52–54]. However, our results prompt the need for monitoring of vitamin D after administration, and day 3 seems to be reasonable since the absorption of vitamin D is expected to be complete after 48 h. Further investigation will have to evaluate whether patients unable to increase their plasma levels after 3 days may benefit from another loading dose.

Indeed, vitamin D supplementation effect may be influenced by some patient characteristics, and in our cohort, the factors influencing negatively the impact of vitamin D on survival were female gender, surgical admission, and history of chronic kidney disease and chronic liver disease. Female gender is a known contributing factor for hypovitaminosis D in the general population in post menopausal age, but this subject has not been explored in critically ill patients [55, 56]. Surgical patients, in particular for gastrointestinal surgery, are the category most at risk of low vitamin D level, and in this group, several studies have highlighted a predictive ability of patient-centered outcomes by 25-hydroxyvitamin D plasma levels [57–59]. Finally, kidney disease and liver disease likely impacted the survival since they are necessary for vitamin D metabolism. We therefore hypothesize that patients with kidney disease may benefit from adding active vitamin D (i.e., calcitriol) to cholecalciferol [60]. The results of the multiple analysis, where vitamin D supplementation does not remain statistically significant in reducing 28-day mortality when strong mortality determinants are added to the model (age, BMI, comorbidities, and mechanical ventilation at admission), suggest that if indeed vitamin D supplementation can lower mortality risk, its effect is smaller than these well-known factors. However, all these factors are non-modifiable, therefore even a modest benefit may be clinically relevant and further large prospective studies are warranted.

Our study has some limitations. First, as this was a post hoc analysis not predefined in the trial protocol, the results come with an increased risk of bias. Second, since the results are based on a subgroup after exclusion of (even limited and for a rational reason) a number of patients, our positive findings of reduction in mortality through vitamin D_3_ on univariate analysis should be considered explorative and hypothesis-generating and need to be confirmed in prospective randomized controlled trials.

Despite these limitations, we believe our study has a number of strengths. First, this study uses data collected as part of the VITdAL-ICU trial, designed, and conducted with high methodological standards. The trial was designed relying on a strong pathophysiological basis and preliminary observational data: the inclusion criteria created a quite homogeneous cohort, the randomization process was very effective, and the data collection was extensive, with few missing data. Further, there is a strong biological and epidemiological rationale for the hypothesis explored (time lag between vitamin D administration and effective biological effects). Finally, we utilized several statistical tests to confirm the relationship between vitamin D supplementation, vitamin D levels, covariate, and patient outcome.

## Conclusions

In this largest RCT undertaken on high-dose vitamin D supplementation in critically ill patients, there was an association between treatment and reduction in 28-day mortality when we removed patients who died or were discharged early, but this effect was not confirmed independently when adjusted for factors associated with mortality. The detailed results may be of help when designing future RCTs and selecting a reasonable target population. The increase in vitamin D level on day 3 was associated with a survival benefit in patients with baseline deficiency. In anticipation of more evidence from bench research about the pathophysiology of vitamin D and biomarkers of its late effects in acute severe illness, the two unpublished large RCTs (VITDALIZE, NCT03188796 and VIOLET, NCT03096314) will further elucidate the potential role of vitamin D supplementation in improving clinical outcomes in critical illness.

## Additional file

Additional file 1: **Table S1.** Effect of vitamin D treatment on different mortality outcomes in population included in the secondary analysis excluding early deaths, 432 patients: 28-day mortality, ICU mortality, hospital mortality, and 6-month mortality. Table S2. Effect of vitamin D treatment on different mortality outcomes in the intention-to-treat population, 475 patients: 28-day mortality, ICU mortality, hospital mortality, and 6-month mortality. (DOCX 14 kb)

## Abbreviations

1,25(OH)D: 1,25-Dihydroxyvitamin D
25(OH)D: 25 Hydroxyvitamin D
BMI: Body mass index
SAPS 2: Simplified acute physiology score 2
TISS: Therapeutic intervention scoring system
VDBP: Vitamin D-binding protein
