# Supplementary material for: Trying to identify who may benefit most from future vitamin D intervention trials: a post hoc analysis from the VITDAL-ICU study excluding the early deaths
Source: Crit Care. 2019 Jun 4;23:200. doi: 10.1186/s13054-019-2472-z (PMC6549317; doi:10.1186/s13054-019-2472-z)
Supplement: Supplementary file 1 — Table S1. Effect of vitamin D treatment on different mortality outcomes in population included in the secondary analysis excluding early deaths, 432 patients: 28-day mortality, ICU mortality, hospital mortality, and 6-month mortality. Table S2. Effect of vitamin D treatment on different mortality outcomes in the intention-to-treat population, 475 patients: 28-day mortality, ICU mortality, hospital mortality, and 6-month mortality. (DOCX 14 kb) [file 13054_2019_2472_MOESM1_ESM.docx]

**Table S1.** Effect of Vitamin D treatment on different mortality outcomes in population included in the secondary analysis excluding early deaths and early discharge (ICU stay less than 7 days), 410 patients: 28-Day Mortality, ICU Mortality, Hospital Mortality, 6-month Mortality.

| **Mortality Outcomes** | **Placebo (n = 206)**  **N (%)** | **Vitamin D_3_ (n = 204)**  **N (%)** | **OR (95% CL)** | **P Value** |
| --- | --- | --- | --- | --- |
| **28-Day Mortality** | 47 (22.8) | 30 (14.7) | 0.58 (0.35-0.97) | 0.035 |
| **ICU Mortality** | 44 (21.4) | 33 (16.2) | 0.71 (0.43-1.17) | 0.179 |
| **Hospital Mortality** | 63 (30.6) | 45 (22.1) | 0.64 (0.41-1.00) | 0.050 |
| **6-Month Mortality** | 79 (38.4) | 60 (29.4) | 0.67 (0.44-1.01) | 0.055 |

**Table S2.** Effect of Vitamin D treatment on different mortality outcomes in the intention-to-treat population 475 patients: 28-Day Mortality, ICU Mortality, Hospital Mortality, 6-month Mortality.

| **28-Day Mortality** | | | |
| --- | --- | --- | --- |
|  | **Alive, N (%)** | **Deceased, N (%)** | **P Value** |
| **Vitamin D,** N= 237 | 185 (78.1) | 52 (21.9) | 0.14 |
| **Placebo,** N= 238 | 170 (71.4) | 68 (28.6) |  |
| **ICU Mortality** | | | |
| **Vitamin D,** N= 237 | 183 (77.2) | 54 (22.8) | 0.86 |
| **Placebo,** N= 238 | 175 (73.5) | 63 (26.5) |  |
| **Hospital Mortality** | | | |
| **Vitamin D,** N= 237 | 170 (71.7) | 67 (28.3) | 0.18 |
| **Placebo,** N= 238 | 153 (64.7) | 84 (35.3) |  |
| **6-Months Mortality** | | | |
| **Vitamin D,** N= 237 | 154 (65.0) | 83 (35.0) | 0.09 |
| **Placebo,** N= 238 | 136 (57.1) | 102 (42.9) |  |
